# Pregnancy after kidney transplantation: 40 years single-center experience

**DOI:** 10.1590/2175-8239-JBN-2023-0061en

**Published:** 2023-12-08

**Authors:** Eloísa Radaelli, Gisele Meinerz, Lázaro Pereira Jacobina, Rosana Mussoi Bruno, Juliana Alves Manhães de Andrade, Valter Duro Garcia, Elizete Keitel

**Affiliations:** 1 Universidade Federal de Ciências da Saúde de Porto Alegre, Programa de Pós-Graduação em Patologia, Porto Alegre, RS, Brazil.; 2 Santa Casa de Misericórdia de Porto Alegre, Porto Alegre, RS, Brazil.

**Keywords:** Pregnancy, Kidney Transplantation, Pregnancy Outcomes, Gestação, Transplante Renal, Desfechos Gestacionais

## Abstract

**Background:**

Kidney transplantation (KT) improves quality of life, including fertility recovery.

**Objective:**

to describe outcomes of post-KT pregnancy and long-term patient and graft survival compared to a matched control group of female KT recipients who did not conceive.

**Methods:**

retrospective single-center case-control study with female KT recipients from 1977 to 2016, followed-up until 2019.

**Results:**

there were 1,253 female KT patients of childbearing age in the study period: 78 (6.2%) pregnant women (cases), with a total of 97 gestations. The median time from KT to conception was 53.0 (21.5 – 91.0) months. Abortion rate was 41% (spontaneous 21.6%, therapeutic 19.6%), preterm delivery, 32%, and at term delivery, 24%. Pre-eclampsia (PE) occurred in 42% of pregnancies that reached at least 20 weeks. The presence of 2 or more risk factors for poor pregnancy outcomes was significantly associated with abortions [OR 3.33 (95%CI 1.43 – 7.75), p = 0.007] and with kidney graft loss in 2 years. The matched control group of 78 female KT patients was comparable on baseline creatinine [1.2 (1.0 – 1.5) mg/dL in both groups, p = 0.95] and urine protein-to-creatinine ratio (UPCR) [0.27 (0.15 – 0.44) vs. 0.24 (0.02 – 0.30), p = 0.06]. Graft survival was higher in cases than in controls in 5 years (85.6% vs 71.5%, p = 0.012) and 10 years (71.9% vs 55.0%, p = 0.012) of follow-up.

**Conclusion:**

pregnancy can be successful after KT, but there are high rates of abortions and preterm deliveries. Pre-conception counseling is necessary, and should include ethical aspects.

## Resumo

**Histórico:**

Transplante renal (TR) melhora qualidade de vida, incluindo recuperação da fertilidade.

**Objetivo:**

descrever desfechos gestacionais pós-TR e sobrevida de longo prazo da paciente e do enxerto renal comparada a um grupo controle pareado de receptoras de TR que não conceberam.

**Métodos:**

estudo retrospectivo caso-controle com receptoras de TR de 1977 a 2016, acompanhadas até 2019.

**Resultados:**

foram identificadas 1.253 receptoras de TR em idade fértil no período do estudo: 78 (6,2%) gestantes (casos), total de 97 gestações. Tempo mediano entre TR até concepção foi 53,0 (21,5 – 91,0) meses. Taxa de aborto foi 41% (espontâneo 21,6%, terapêutico 19,6%), parto prematuro, 32%, e a termo, 24%. Pré-eclâmpsia (PE) ocorreu em 42% das gestações que alcançaram pelo menos 20 semanas. Presença de 2 ou mais fatores de risco para desfechos gestacionais desfavoráveis foi significativamente associada a abortos [OR 3,33 (IC95% 1,43 – 7,75), p = 0,007] e perda de enxerto renal em 2 anos. O grupo controle de 78 mulheres com TR foi comparável na creatinina basal [1,2 (1,0 – 1,5) mg/dL nos dois grupos, p = 0,95] e na relação proteína/creatinina urinária (RPCU) [0,27 (0,15 – 0,44) vs. 0,24 (0,02 – 0,30), p = 0,06]. Sobrevida do enxerto foi maior nos casos que nos controles em 5 anos (85,6% vs. 71,5%, p = 0,012) e 10 anos (71,9% vs. 55,0%, p = 0,012) de acompanhamento.

**Conclusão:**

a gestação pode ser bem-sucedida após TR, mas existem altas taxas de abortos e partos prematuros. Aconselhamento pré-concepção é necessário e deve incluir aspectos éticos.

## Introduction

Kidney transplant (KT) is considered the treatment of choice for end-stage chronic kidney disease (CKD)^1^. Brazil ranks 4^th^ in the world for the absolute number of KT^2^, and Santa Casa de Misericórdia in Porto Alegre is one of the largest centers, with over 5,000 procedures performed since the program began in 1977 up to 2019. Over 2,000 of the KT recipients were women, many of them of childbearing age.

In addition to the general improvement in quality of life, women often regain fertility and are able to become pregnant shortly^3,4^. The first successful pregnancy in a KT recipient occurred in 1958 and was published in 1963^5^ and, since then, many more cases were reported. Nevertheless, the incidence of complications is not negligible^6^. Fetal viability, maternal complications, and associated factors are still understudied and underreported subjects^6^.

Maternal complications include pregnancy-related hypertension, pre-eclampsia (PE), eclampsia, gestational diabetes, graft rejection, and infections^6^. Graft rejection is associated with high baseline serum creatinine and fluctuating immunosuppressant levels caused by pregnancy-related changes in volume distribution and renal clearance of these agents^7^.

Miscarriages, prematurity (birth before completing 37 weeks of pregnancy), fetal loss, low birth weight (< 2,500g) and intrauterine growth restriction are more common in transplant patients, although the risk magnitude has not yet been clearly quantified^6,8^.

The ideal conditions for pregnancy after KT are adequate graft function (creatinine < 1.5mg/dL or eGFR > 60mL/min), no or minimal proteinuria (< 500 mg/day), normotension or controlled blood pressure with one drug, no acute rejection episodes in the previous year, good compliance, low and stable immunosuppression dosage, and at least one year after transplantation^6,9–11^. Potentially teratogenic drugs such as mycophenolic acid and mammalian target of rapamycin inhibitors (mTORi) should be withheld for at least 6 to 12 weeks before conception^6,11^.

Most data on post-transplant pregnancy outcomes derive from retrospective observational studies, case series, or national registries (mainly from UK, Europe, and US). McKay and Josephson^11^ compiled data of such registries in 2006, finding an average of 12% miscarriages, 2% stillbirths, 50% preterm deliveries, 50% cesarean sections, and over 50% low birth weight. Among KT recipients, PE rates were about 30%, graft loss in 2 years was 4–14%, and live births 71–78%. More recently, the Transplant Pregnancy Registry International reported their 30-year data collection on 1,251 KT recipients, with 2,233 pregnancies and 26% of fetal losses^12^.

Several studies describe the adverse effects of pregnancy for women and their fetuses; however, few compare long term patient and graft survival of pregnant vs. non-pregnant KT recipients^11^.

This study aims to describe the characteristics of KT recipients at our center that conceived, pregnancy-related complications and outcomes, and to compare their long-term graft and patient survival to matched female controls.

## Methods

This is a retrospective case-control study conducted at the Kidney Transplant Service of Santa Casa de Misericórdia in Porto Alegre, Brazil. Female patients who underwent KT from 1977 to 2016 and were of childbearing age during follow-up were included. Follow-up was set until date of death, return to dialysis, new transplant, or December 31st, 2019, whichever came first. Exclusion criteria were combined kidney and other solid organ transplants except pancreas and follow-up shorter than six months.

Patients that became pregnant (cases) were compared with female controls that did not conceive during follow-up matched for age, donor type, date of transplant, and immunological risk.

Data collected for cases included time from transplantation to pregnancy, baseline graft function and urinary protein-to-creatinine ratio (UPCR), pre-existent hypertension, and baseline immunosuppressive therapy. Maternal complications (PE, eclampsia, gestational hypertension, and diabetes) and pregnancy outcomes (spontaneous abortion, therapeutic abortion, premature or term delivery, stillbirth, delivery type, birth weight) were recorded. After pregnancy, the number of acute rejection episodes and kidney function at 6 and 12 months were registered.

The number of risk factors for poor pregnancy outcomes were computed, and defined as creatinine > 1.5mg/dL or eGFR < 60 mL/min, UPCR > 0.5, pre-existing hypertension and conception within the first year post-transplantation.

Control group data included baseline immunosuppression, creatinine, UPCR, hypertension, and rejection episodes.

Patients were also separated in two periods according to the standard immunosuppressive maintenance therapy: before the year 2000, when cyclosporine and azathioprine were the regimen of choice, and after 2000, with tacrolimus and mycophenolic acid as the main agents.

When pregnancy desire was manifested or unplanned conception was acknowledged, we switched immunosuppression from mycophenolic acid or mTORi to azathioprine as standard care, adjusting calcineurin inhibitors according to trough levels and maintaining low steroid dose.

Based on a previous study at our center^13^, the sample size was calculated assuming a 10% graft survival difference in 5 years after transplantation between patients that did and did not become pregnant. We had a power of 77.3%, with a significance level of 5% (two-tailed) to detect this difference in a 1:1 case-control study with 65 patients in each group. The calculation was performed using the WinPepi program, version 3.85 (Power of test for difference between proportions – module P1). The study was approved by the hospital ethics committee.

Data gathered from medical records were analyzed using the Statistical Package for Social Sciences (SPSS®) version 21.0 (SPSS INC., Chicago, IL). Continuous variables with normal distribution are presented as mean and standard deviation and compared with parametric tests. Variables with non-normal distribution are presented as median and 25–75% interquartile interval and compared with non-parametrical tests. Categorical variables are presented as absolute and relative frequencies and compared with chi-square. Comparisons between groups were performed using the Mann-Whitney U test. Intragroup comparisons were performed using the Wilcoxon paired test. Patient and graft survival were assessed with Kaplan-Meier. ROC analysis was performed to identify the most sensitive and specific creatinine, eGFR and UPCR values associated with 2-year post-pregnancy graft loss. A significance level of p < 0.05 was considered for all analyses.

## Results

During the study, there were 1,253 female kidney transplant recipients of childbearing age. In the first period (1977 to 1999), 44 (12.9%) out of 341 women conceived. In the second period (2000 to 2016), 34 (3.7%) of 912 women became pregnant. Sixty-six (84.6%) patients conceived once, and 12 had more than one pregnancy, resulting in a total of 97 gestations analyzed.

Table 1 shows the baseline characteristics of 78 cases and 78 controls. There were no significant differences between groups, including baseline kidney function and UPCR. There was one case with type 1 diabetes and two controls, and the case received a simultaneous kidney and pancreas transplant.

**Table 1:** Baseline characteristics of cases and controls

Variable	Cases n = 78	Controls n = 78
Median age at transplant^a^	27.0 (22 – 32)	24.0 (17 – 28.2)
Living donor (%)	54 (69)	54 (69)
Fist Transplant (%)	70 (89)	71 (91)
Diabetes (%)	1	2
**Transplant period**		
1977–1999	44	44
2000–2016	34	34
**Induction therapy**	15	15
Anti-CD3	1	1
Thymoglobulin	4	4
Anti-IL2 Receptor	10	10
**Maintenance Immunosuppression**		
Azathioprine	59	57
Mychophenolic acid	17	17
Cyclosporine	45	42
Tacrolimus	18	20
Steroids	74	77
mTOR inhibitors	1	0
Serum creatinine, mg/dL^a^	1.2 (1.0 – 1.5)	1.2 (1.0 – 1.5)
eGFR, mL/min/1.73m^2 a^	63.0 (47 – 77)	63.0 (45.7 – 78.5)
UPCR^a^	0.27 (0.15 – 0.44)	0.24 (0.02 – 0.30)

Legend: eGFR: estimated glomerular filtration rate using CKD-EPI calculator. UPCR: urine protein-to-creatinine ratio.

a Values presented in median (interquartile interval, 25–75)

Table 2 presents data related to 78 cases and 97 pregnancy outcomes. Median time from transplantation to conception was 53.0 (21.5 – 91.0) months. Median creatinine before conception and one year after did not differ [1.2 (1.0–1.5) mg/dL vs. 1.2 (1.1–1.5) mg/dL, p = 0.114]. Median UPCR before pregnancy and one year after did not differ [0.3 (0.16 – 0.6) vs. 0.29 (0.14 – 0.80), p = 0.225]. Sixty-five (67%) cases had preexisting hypertension and 43 (44.3%) had more than one risk factor for poor pregnancy outcomes. Fifty-seven (58.7%) patients reached at least 20 weeks of pregnancy, 23 (40.3%) of them had PE and one had eclampsia and died of related complications.

**Table 2:** Pregnancy data and outcomes

	Patients n = 78 Pregnancies n = 97
**Time between transplantation and conception, months** ^a^	53.0 (21.5 – 91.0)
**Number of pregnancies, n (%)**	
1	66 (68)
2	7 (7.2)
3	3 (3.0)
4	2 (2.0)
**Number of risk factors for poor pregnancy outcomes, n (%)**	
0	21 (21.6)
1	32 (32.9)
2	26 (26.8)
3	16 (16.4)
4	2 (2.0)
**Transplant period 1977–1999**	**56 (57.7)**
Cyclosporine	36 (64.2)
Azathioprine	56 (100)
**Transplant period 2000–2016**	**41 (42.2)**
Cyclosporine	17 (41.4)
Azathioprine	18 (43.9)
Tacrolimus	22 (53.6)
Mycophenolate	22 (53.6)
mTOR inhibitors	1 (2.4)
**Abortions n (%)**	40 (41.2)
Spontaneous	21 (21.6)
Therapeutic	19 (19.6)
**Stillbirths n (%)**	2 (2.1)
**Live births n (%)**	55 (56.7)
Preterm	31 (32.0)
At term	24 (24.7)
**Preeclampsia/eclampsia, n (%)**	24^b^/57^c^ (42.1)
Cesarian section, n (%)	47/57^c^ (82.4)
Weight at birth (g)^a^	2,540 (1,950 – 2,750)
**Serum creatinine, mg/dL** ^a^	
6 months after pregnancy	1.2 (1.0 – 1.5)
12 months after pregnancy	1.2 (1.1 – 1.5)
**eGFR, mL/min/1.73m** ^2 a^	
6 months after pregnancy	63.0 (47 – 77)
12 months after pregnancy	63.0 (48 – 77)
**Proteinuria after pregnancy (UPCR)** ^a^	0.29 (0.14 – 0.80)
Post-pregnancy acute graft rejection, n (%)	12 (12.3)
Post-pregnancy graft loss within 2 years, n (%)	17 (21.7)

Legend: eGFR, estimated glomerular filtration rate using CKD-EPI calculator. UPCR, urine protein to creatinine ratio.

a Values presented in median (interquartile interval, 25–75)

b One patient had eclampsia

c Number relative to pregnancies that completed at least 20 weeks

Fifty-five (56.7%) pregnancies resulted in live births, 31 (54.3%) of them were preterm. There were 2 (2.0%) stillbirths and 40 (41.2%) abortions (spontaneous = 21, therapeutic = 19), and these were associated with the presence of two or more risk factors for adverse outcomes [OR 3.33 (95%CI 1.43 – 7.75), p = 0.007]. Regarding therapeutic abortions, they were more frequent in the first period [(KT before 2000), 16 vs. 3, p = 0.010], all patients had pre-existing hypertension, and 12 (63.1%) had baseline UPCR > 0.5. Regarding delivery route, there were 6 (10.9%) vaginal deliveries, 5 of them after 2000. For the 47 patients that had cesarean sections, 26 (55.3%) had preexisting hypertension, 21 (44.6%) had pre-eclampsia, and 27 (57.5%) were preterm deliveries. There were no associations of immunosuppressant regimens and pregnancy outcomes.

**Figure 1.**
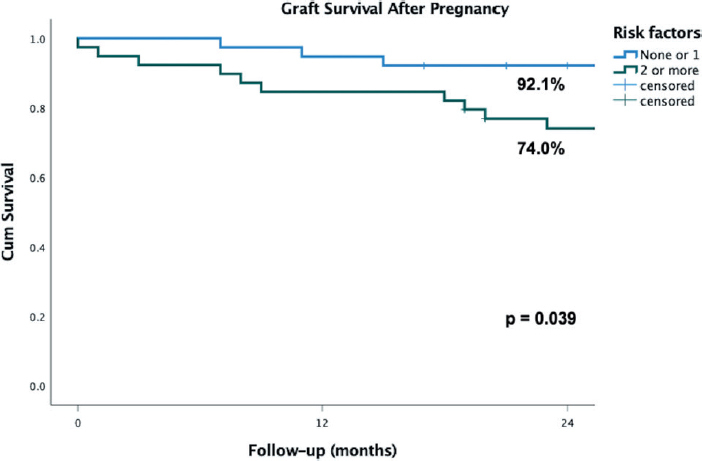
Graft survival of female kidney transplant recipients in a 2-year follow-up after pregnancy, according to number of risk factors for poor pregnancy outcomes.

There were 12 (12.3%) events of acute graft rejection post-pregnancy. The one patient that received a simultaneous kidney and pancreas transplant did not experience disfunction of neither graft. There were 14 (17.9%) graft losses within 2 years of pregnancy, and they were associated presence of two or more risk factors for poor pregnancy outcomes [OR 4.42 (95% CI 1.12 – 17.3), p = 0.037]. Figure 1 shows graft survival according to the sum of these risk factors.

**Figure 2.**
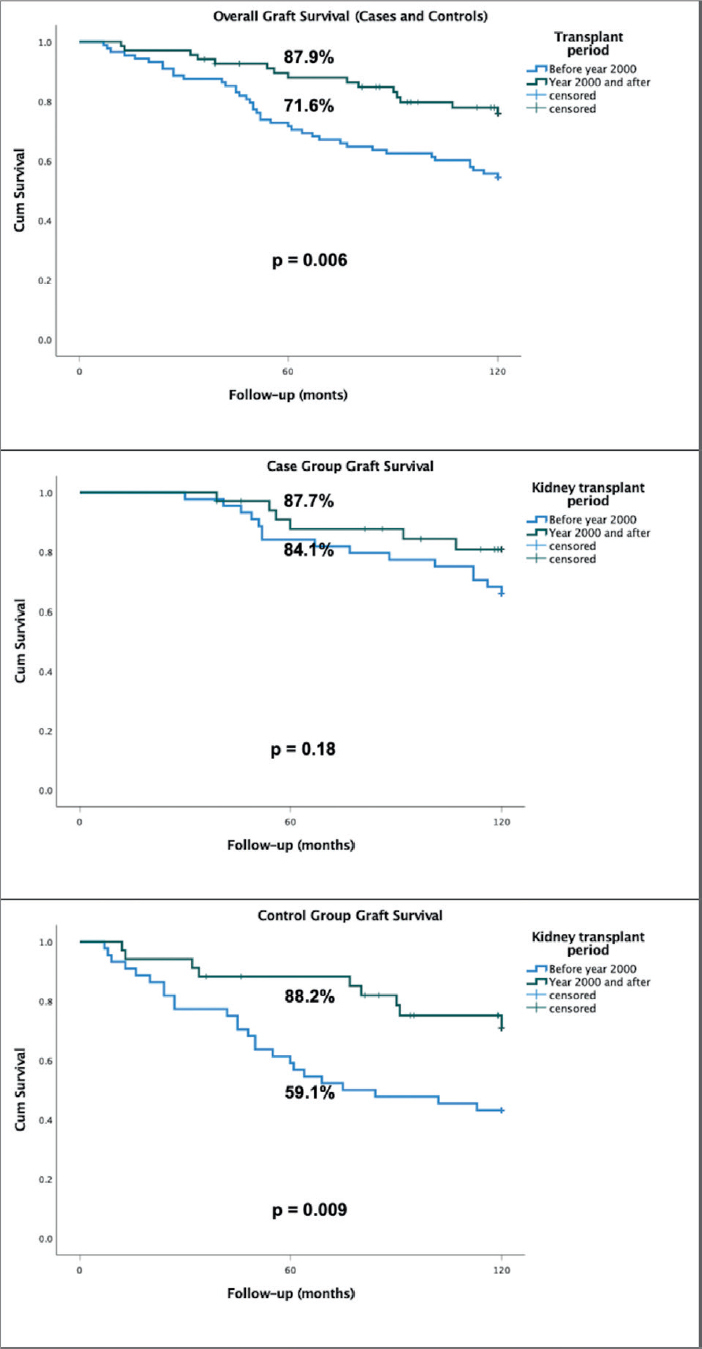
Graft survival according to period of kidney transplantation, before or after 2000, of cases plus controls (top panel), cases (mid panel) and controls (bottom panel).

There were no differences in 5-year graft survival of cases according to the transplant period (before or after 2000) (87.7% vs. 84.1%, p = 0.18). Patients transplanted before 2000 (cases plus controls) had a significantly lower overall graft survival in 5 years of follow-up than those transplanted after 2000 (71.6% vs. 87.9%, p = 0.006). These data are shown in Figure 2.

**Figure 3.**
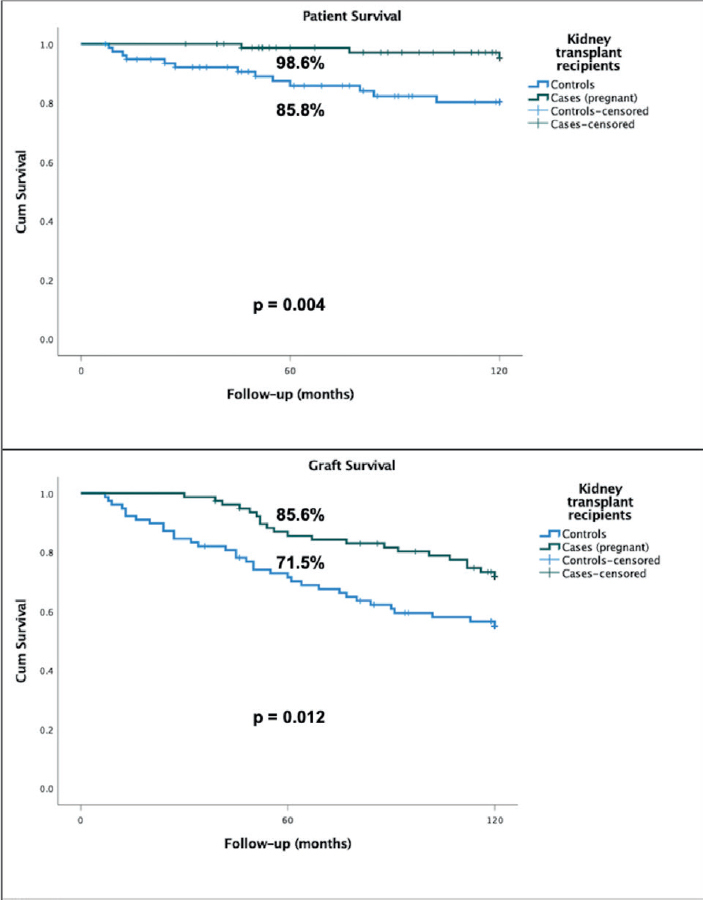
Patient (top panel) and graft (bottom panel) survival of female kidney transplant recipients that did (cases) and did not (controls) conceive during follow-up.

Graft survival after transplantation was higher in cases compared to controls in 5 years (85.6% vs 71.5%, p= 0.012) and 10 years (71.9% vs 55.0%, p = 0.012) of follow-up. Patient survival was also higher in cases than controls in 5 years (98.6% vs 85.8%, p = 0.004) and 10 years (95.2% vs 80.3%, p = 0.004) of follow-up. These data are shown in Figure 3. Cox-regression analysis showed that transplantation before 2000 [HR 2.36 (95% CI 1.3 – 4.2), p = 0.005] and control group [HR 2.07, (95%CI 1.2 – 3.5), p = 0.09] were independently associated with lower 10-year graft survival.

The area under the receiver operating characteristic (ROC) curve (AUC) analysis was performed to evaluate accuracy of baseline variables in predicting 2-year post-pregnancy graft survival. For creatinine, the AUC was 72.7% (95% CI 0.59 – 0.85, p = 0.001), and a value of 1.55 mg/dL provided 58.8% sensitivity with 85% specificity. For eGFR, the AUC was 71.3% (95% CI 0.57 – 0.84, p = 0.002), and 60.5 mL/min/1.73m^2^ provided 64.7% sensitivity with 64% specificity. For UPCR, the AUC was 79.5% (95% CI 0.66 – 0.92, p < 0.0001) and a value of 0.51 provided 75% sensitivity with 84% specificity.

## Discussion

Fertility recovery is related to improved overall health after KT. However, post-KT pregnancy is not risk-free, and patients and their families should be advised of possible maternal and fetal outcomes. In our study, the proportion of pregnancies decreased over the years, which is probably associated with our multidisciplinary and active counseling and a consequent reduction in non-planned conceptions.

It has been more than 50 years since the first successful post-transplant pregnancy was described^5^, and since then many cases more were reported^14^. Given that motherhood was feasible for these patients, efforts were made to understand and mitigate the risks to both mother and child. The difficulties in gathering good quality evidence lie in the heterogeneity of the population, the lack of large studies, and the inconsistency of nomenclature^11^. The depiction of the ideal candidate for post-transplant pregnancy is somewhat well established, but the impact that less-than-ideal conditions may have on the outcomes remains non-quantifiable^6^.

The risk factors for poor pregnancy outcomes are defined based on observational studies and best practice guidelines^6,10,15^, compiling information from retrospective studies, transplant registries, and case series^16^. Establishing optimal cut-off values for creatinine, eGFR, protein excretion, blood pressure, and timing is complex, and an individualized approach is advised^6^.

In our sample of 97 pregnancies in 78 women, only 21% had none of the described risk factors prior to pregnancy. The median time from transplantation to conception was 53 months. Recent consensus reports have reduced the recommended interval from two years to one year, in view of stable graft function and immunosuppression and generally lower risk of rejection with newer and more potent treatments^10,11,17,18^.

Baseline graft function is considered one of the most important prognostic factors for pregnancy and graft outcomes in KT patients^19^. The risk of graft loss is considered low when baseline kidney function is normal, but the definition of normal is not clear^6^. Graft disfunction assessment during pregnancy is challenging, since modifications in volume distribution and hyper-filtration usually reduce creatinine levels. The median creatinine in our case group was below 1.5 mg/dL at baseline and remained stable at 6 and 12 months after pregnancy. The median UPCR was below 0.5 mg/day and also remained stable at 1-year follow-up. These values are consistent with the literature and may have influenced the 2-year graft survival and the rate of post-pregnancy acute rejection episodes. ROC analysis showed that UPCR above 0.51 was a good predictor of graft loss in 24 months in our study.

It was previously believed that immunological changes for fetus tolerance during pregnancy contributed to a lower incidence of rejections during this period. However, this is counterbalanced by maintenance immunosuppression modifications and fluctuating trough levels, and rejection rates are about 2–12%^11,14,20^.

In the general population, hypertensive disorders complicate around 10% of pregnancies, up to half of them with PE^6^. The prevalence of hypertension in pregnant women with CKD ranges from 27% to 40% and CKD is an independent risk factor for adverse pregnancy outcomes. Women with CKD are ten times more likely to have PE compared to women without kidney disease. In KT patients, PE occurs in up to 40% of pregnancies^11,13,21,22^, similar to our findings. It is important to note that in the single case of eclampsia in our sample, the patient did not have any risk factor prior to conception and eventually died of related complications. This highlights the importance of sharing patient care with a high-risk prenatal team.

Hypertension and proteinuria combined are thought to exponentially influence adverse events^6^. The presence of 2 or more risk factors in our population was significantly associated with abortions and graft losses within 2 years of pregnancy, and the strongest association was with hypertension and proteinuria.

Abortion occurs in up to 26–35% of post-transplant pregnancies and may be associated with high rate of maternal risk factors and the use of immunosuppressive medication^13,14,23,24^. Our rate was higher (40%), and therapeutic terminations were more frequent in patients transplanted before 2000. This is possibly related to the fact that less information on patient survival and graft function was available at that time, as well as concerns about malformations and obstetric complications. Of note, all patients that underwent therapeutic terminations had preexisting hypertension and most had baseline proteinuria. Therapeutic abortions were more frequent in South America compared to other countries in a recently published meta-analysis that reviewed over 6,000 pregnancies^14^. Although after 2000, with more frequent mycophenolate exposure, an association with miscarriage could be speculated.

The main reported fetal outcomes are preterm delivery, restricted intrauterine growth, and low birth weight^11,25,26^. The risk of malformations, except those linked to genetic diseases, is not increased if teratogenic drugs were discontinued at least six weeks before conception^6,25^.

Over half of the pregnancies in our study resulted in live births, and most of them were preterm. This is a lower rate than other studies^14,27^. Prematurity may lead to several consequences that should be addressed in pre-conception counseling, such as perinatal mortality, retinopathy, neurological damage, and the possibility of developing CKD in the future^6^.

There was a very high rate of cesarean sections, similar to other studies^11,14,22^, in which South America had the highest frequency^14^. Delivery route should be determined by obstetric indication, with no impediments to vaginal deliveries. Brazil has a historically high incidence of cesarean deliveries, even in the general population^22^. Since almost all of our patients had cesarean deliveries, we were not able to identify risk factors. Of note, most patients had preexisting hypertension, almost half had pre-eclampsia, and the majority had preterm deliveries.

Graft loss have been reported to be 10% in a 2-year follow-up^14,18^. In our study, 10-year graft survival of was higher in women who were pregnant than in women that never conceived. There was an overall lower graft survival in cases plus controls that received their KT before 2000 compared to transplants that occurred after 2000. This can be explained by advances in the transplant procedure and in immunosuppression in the last decades, ultimately resulting in improved graft function and better quality of life^28^. It could be speculated that women who were able to impregnate had overall better health, culminating with recovered fertility, as demonstrated by similar survival rates in both periods.

The strengths of this study are the large sample size and long follow-up, describing important aspects of post-transplant pregnancies. The weaknesses are those related to limitations of case-control studies and the long time spam that included patients from an era before implementation of electronic medical charts that may have impacted the ability to retrieve accurate information.

Since post-transplant pregnancy has been proven possible and can be successful, the discussion has shifted to ethical aspects opposing^6,25^: a) the autonomy of the woman to decide to become pregnant after full disclosure of the potential risks to herself, her graft, and the future child, to decide to get pregnant, and b) the non-maleficence and social justice principles, that advise against taking unnecessary risks, even though these are currently inconsistent and unquantifiable. Pregnancy is an added value for KT women. Reports like this are important to help build the necessary evidence for best counseling and sharing decisions with the multidisciplinary team.
